# Investigation of Non-Enzymatic Glycosylation of Human Serum Albumin Using Ion Trap-Time of Flight Mass Spectrometry

**DOI:** 10.3390/molecules17088782

**Published:** 2012-07-25

**Authors:** Xue Bai, Zhangjie Wang, Chengcai Huang, Zhe Wang, Lianli Chi

**Affiliations:** 1 National Glycoengineering Research Center, Shandong University, Jinan, Shandong 250100, China; E-Mail: bxsy8011@sina.com; 2 State Key Laboratory of Microbial Technology, Shandong University, Jinan, Shandong 250100, China; E-Mail: wangzhangjie@yahoo.com; 3 Life Science and Clinical Department, Shimadzu International Incorporation, Beijing 100020, China; E-Mail: fxhcc@shimadzu.com.cn; 4 Division of Endocrinology and Metabolism, Provincial Hospital affiliated to Shandong University, Jinan, Shandong 250021, China

**Keywords:** glycation, human serum albumin, ion trap-time of flight, mass spectrometry, peptide fingerprinting, diabetes, incorporation ratio

## Abstract

Non-enzymatic glycosylation or glycation involves covalent attachment of reducing sugar residues to proteins without enzyme participation. Glycation of glucose to human serum albumin *in vivo* is related to diabetes and many other diseases. We present an approach using liquid chromatography coupled to an electrospray ionization source of a hybrid ion trap-time of flight (IT-TOF-MS/MS) tandem mass spectrometer to identify the glycation sites on serum albumin from both a healthy person and a diabetic patient. The MetID software, which is commonly used for screening metabolites, is adapted for peptide fingerprinting based on both *m*/*z* values and isotopic distribution profiles. A total of 21 glycation sites from the healthy person and 16 glycation sites from the diabetic patient were identified successfully. We also demonstrate the use of matrix assisted laser desorption ionization-time of flight mass spectrometry to estimate the incorporation ratio of glucose to albumin during glycation. Results from this study show that the glycation in healthy person is more complicated than previously thought. Further analysis of incorporation ratio distribution may be necessary to accurately reflect the change of serum albumin glycation in diabetic patients.

## 1. Introduction

It is well known in the literature that enzymes are involved in the glycosylation process, as exemplified in the *N*-glycosylation at asparagine residues, the *O*-glycosylation at threonine or serine residues and the glycosylphosphatidylinositol-anchoring at the C-terminus of some proteins. There is also a category of glycosylations that occurs without the participation of enzymes. Such glycosylation is named non-enzymatic glycosylation or glycation, in which reducing sugars are covalently attached to the lysine, arginine or cysteine residues of proteins [1]. The reducing carbonyl group of glucose can react with the amine groups of human serum albumin (HSA) to form glycated HSA (GA) (Schiff-base) which results in a 162 Da molecular weight increase for each glucose-induced glycation on HSA. This is followed by the Schiff-base reorganized itself to the more stable aminomethyl ketone by the Amadori rearrangement [2].

Glycation has significant impact on the conformation and function of HSA. The tertiary structure conformation was revealed to be affected by glycation using circular dichroism, fluorescence, and microviscometer [3]. Binding affinity of GA to protein hemin and long chain fatty acid cis-parinaric acid decreased 1-20 folds compared to the unmodified HSA (NGA) [4]. *In vivo*, the Amadori reaction product of glycation can undergo Maillard reactions and other more complicated reactions to form advanced glycation end products. Advanced glycation end products are implicated in many severe diseases such as cancer, cardiovascular disease, and Alzheimer’s disease [5,6,7]. The early stage glycation draws increasing attentions in many aspects not only because it is the first step of glycation, but also it is considered as a valuable biomarker for controlling diabetes [8,9].

The GA approximately accounts for 6–15% of total HSA in healthy persons [2], while the proportion may increase two- to three-fold in diabetic patients [1]. Many analytical methods have been established to measure the GA level in serum, including affinity chromatography, post-column fluorescence derivatization-high performance liquid chromatography (HPLC), enzyme linked boronate immunoassay, and colorimetric methods [10,11,12,13,14]. The affinity chromatography method can also be used to separate GA from NGA by specific binding between the carbohydrate moiety in GA and boronate groups immobilized on the resin [15]. Then the purified GA can be used for detailed structural study, for example, glycation site determination.

The common strategy for glycation site determination is to analyze the peptide digests from GA. In earlier studies, the glycation site-containing peptides were labeled by reduction with NaB^3^H_3_, purified by affinity HPLC and reversed phase HPLC with radioactivity monitor, and then identified by amino acid composition analysis [16,17]. Obviously this approach was cumbersome and time-consuming. Mass spectrometry (MS), a widely used technique in protein structure and modification studies, facilitated the glycation site determination of GA. Liquid chromatography (LC)-electrospray ionization (ESI)-ion trap MS, capillary zone electrophoresis-ESI-ion trap MS, and matrix assisted laser desorption ionization-time of flight (MALDI-TOF) MS were reported for successful study the glycation of albumin *in vitro* [12,18,19]. To determine endogenous glycation sites, extraction of HSA from serum and separation of GA from NGA steps were necessary before MS analysis. Quadrupole-time of flight (Q-TOF), one the most popular types of MS analyzer in peptide mapping fields, was reported to be applied in glycation sites study by different research groups [20,21]. A total of 29 potential glycation sites of GA from diabetic patients were revealed by previous reports while variation across these research results were noticed [16,20,21].

Ion trap-time of flight (IT-TOF) is a hybrid mass analyzer that combines the high sensitivity of ion trap and fast scan speed of TOF. It also has the capability of multiple stage tandem MS (MS/MS^n^) with relatively high resolution and mass accuracy. With these advantages, IT-TOF has become increasingly popular in protein identification and peptide fingerprinting [22,23,24,25,26]. In this study, we demonstrated the use of IT-TOF for study of glycation sites in GA from both diabetic patient and healthy person. Many glycation sites from previous reports were confirmed. And several novel glycation sites were discovered as well. In addition, the ratio of glucose incorporated to HSA, a potentially important character of the GA glycoconjugate which seemed overlooked by previous researchers, was successfully elucidated using MALDI-TOF-MS.

## 2. Results and Discussion

### 2.1. GA Purification and GA Level in Serum

**Figure 1:**
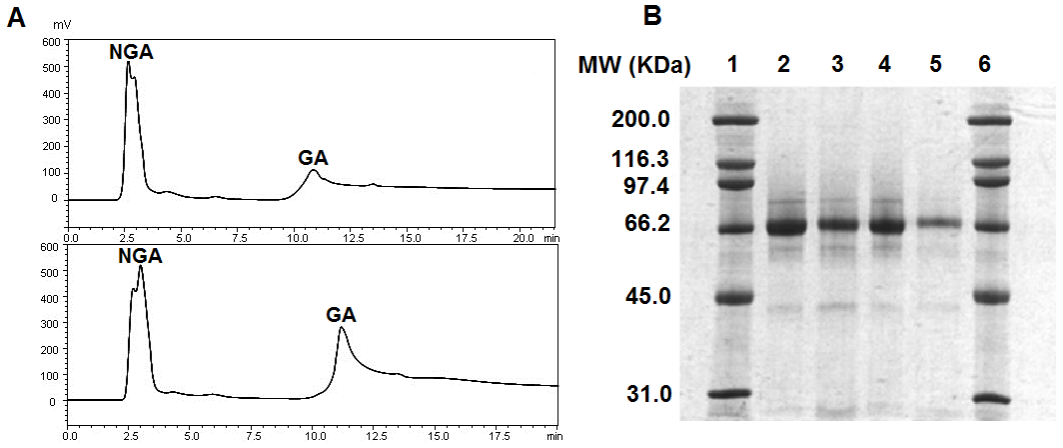
Affinity chromatography and SDS-PAGE analysis of serum albumin. (**A**) Affinity HPLC separation of albumin from healthy person (top) and diabetic patient (bottom). (**B**) SDS-PAGE analysis of albumin after affinity HPLC separation, lane 1. Protein MW marker; lane 2. NGA from healthy person; lane 3. GA from healthy person; lane 4. NGA from diabetic patient; lane 5. GA from diabetic patient; and lane 6. protein MW marker.

Total HSA was firstly extracted from serum by polyethylene glycol (PEG) precipitation. Next, NGA and GA were separated on boronate affinity column. The NGA was eluted without retention while the GA was bound to the column due to the interaction between *cis*-diols from glucose residues and the boronate ligands. Mobile phase containing sorbitol was then used to competitively elute GA. The affinity chromatography separation of GA and NGA was shown in Figure 1A. The chromatogram on the top was from healthy person and the one on the bottom was from diabetic patient. By comparison of GA levels in serum between healthy person and diabetic patient, the percentage of GA in total HSA increased from 10.1% to 26.8%. The significant elevation of GA level was commonly observed for diabetic patients, as a consequence of high blood glucose level [2].

Figure 1B showed SDS-PAGE analysis of GA and NGA samples after affinity chromatography. Lanes 2, 3, 4, and 5 corresponded to NGA from healthy person, GA from healthy person, NGA from diabetic patient, and GA from diabetic patient, respectively, and were bracketed by protein MW markers (lanes 1 and 6). Single major band with MW approximate 66 kDa was revealed for all four samples. A slight increase of relative migration distance was observed for the NGA bands compared to the GA bands, possibly induced by the addition of glucose residues to HSA.

### 2.2. Glycation Site Determination

Peptide mapping by MS (or peptide mass fingerprinting) is a powerful tool to confirm amino acid sequence or find predicted modification on known proteins. Trypsin, which cleaves peptides on the C-terminal side of lysine and arginine amino acid residues, is one of the commonly used endoproteinases to produce peptides for MS analysis. Since the glycation occurred at lysine residues, we also used endoproteinase Glu-C to degrade GA besides of trypsin. High sequence coverage (88% for GA from healthy person and 78% for GA from diabetic patient) was achieved by combining the mapping results of both endoproteinase Glu-C and trypsin digested peptides.

**Figure 2:**
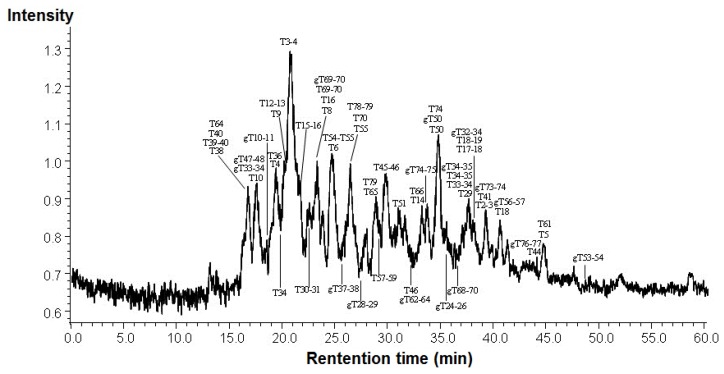
LC-ESI-CID-MS/MS total ion chromatogram of trypsin digested GA from healthy person.

Figure 2 showed the LC/MS/MS total ion chromatography of trypsin digested peptides from GA of the healthy person. Approximate sixty peptides and glycated peptides were separated on reversed phase HPLC, detected by MS, and identified by MetID software.

**Figure 3:**
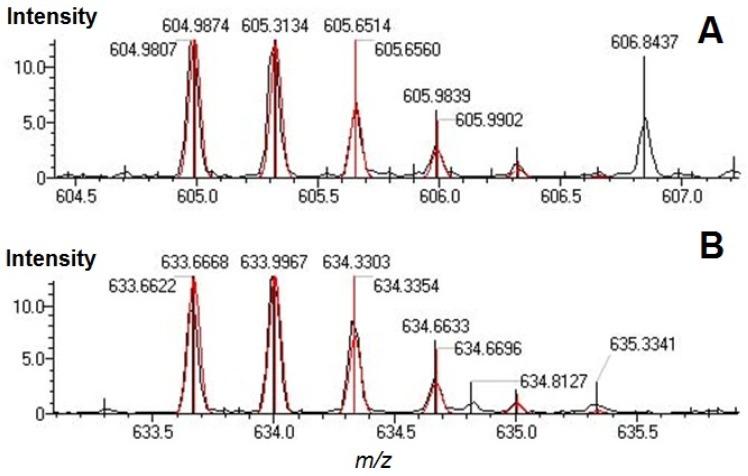
MS profile matching of glycated peptides. (**A**) Glycated peptide gT32-33 (sequence LSQRFPKglcAEFAEVSK). (**B**) glycated peptide gT34-35 (sequence AEFAEVSKglcLVTDLTK).

The MetID software was originally designed for searching metabolites by inputting known parent formula and possible modifications. Here we demonstrated the use of this software for searching protein modifications by inputting C_6_H_10_O_5_ as parent formula and all theoretical enzymatic digested peptides’ formula as modifications. For unmodified peptides, we used C_0_H_0_ as parent formula. Unlike many other peptide mapping softwares that deconvolute experimental peaks then match them to theoretical peptide masses, MetID generates *m*/*z* envelopes according to theoretical chemical formula and then searches experimental peaks based on the value of *m*/*z* and the isotopic distribution. This searching scheme reduced the chance of false matches by avoiding automatic deconvolution process and facilitated the manual checking of original mass spectrum afterwards. Examples of finding glycated peptides using MetID were shown in Figure 3. The red profiles were theoretical *m*/*z* envelopes generated by the software and the black profiles were experimental mass spectrum. The two species were assigned confidently as gT32-33 (sequence LSQRFPKglcAEFAEVSK, in panel A) and gT34-35 (AEFAEVSKglcLVTDLTK, in panel B).

**Figure 4:**
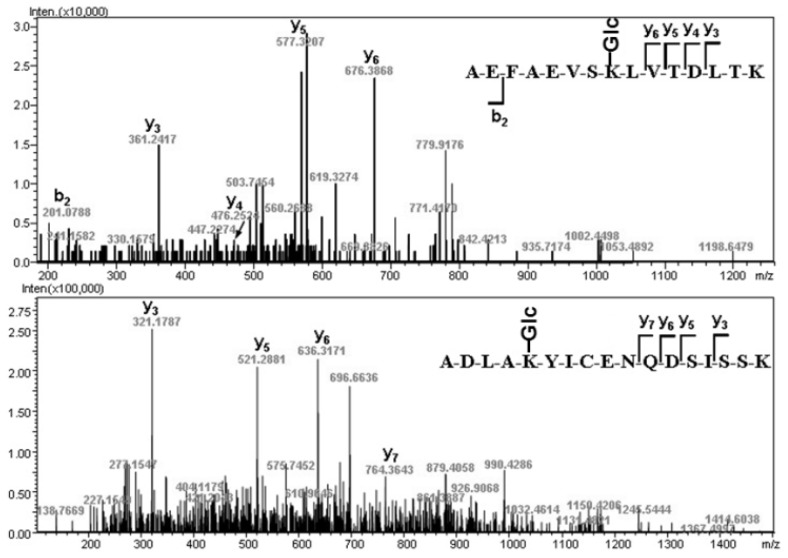
MS/MS analysis of glycated peptides. The top MS/MS spectrum is glycated peptide gT34-35 (sequence AEFAEVSKglcLVTDLTK) and the bottom MS/MS spectrum is glycated peptide gT37-38 (sequence ADLAKglcYICENQDSISSK).

Based on the trypsin experiment (Table 1) and endoproteinase Glu-C experiment (Supplementary Material, Table S1), in total 21 glycation sites were disclosed. It was surprising that there were so many glycation sites in the GA from healthy persons. To confirm the presence of multiple glycation sites, we performed MS/MS analysis on selected ions corresponding to glycated peptides. As the examples shown in Figure 4, glycated peptides gT34-35 (AEFAEVSKglcLVTDLTK) and gT37-38 (ADLAKglcYICENQDSISSK) were indentified unambiguously by their fragment ions in MS/MS. Lys-525 was reported to be the principal glycation site in healthy person, along with a few lysine residues susceptible to be glycated [17,20]. Our results showed that the HSA glycation in healthy person was much more diverse than previously expected. Besides Lys-525, the glycation sites Lys-64, Lys-93, Lys-190 or Lys-195, Lys-205, Lys-225, Lys-233, Lys-262, Lys-274, Lys-281, Lys-323, Lys-351, Lys-378, Lys-413, Lys-432, Lys-475, Lys-545, Lys-557, Lys-564, and Lys-573/Lys-574 were revealed using IT-TOF MS peptide mapping (Table 2). No glycation was found to occur at arginine or cysteine residues.

For the GA from the diabetic patient, 16 glycation sites were revealed based on eight tryptic glycated peptides and 10 endoproteinase Glu-C digested glycated peptides (Supplementary Material, Table S2). Half of these sites (Lys-64, Lys-199, Lys-233, Lys-274, Lys-281, Lys-317, Lys-475, and Lys-525) were reported by other groups previously [16,20,21], and half of them (Lys-190, Lys-225, Lys-240, Lys-286, Lys-323, Lys-372, Lys-413, and Lys-557/Lys-560/Lys-564) were newly discovered (Table 2). Among these sites, only Lys-233 and Lys-525 were claimed to be glycated by all three references and us. Besides the different analytical techniques used in glycation site studies, the variation might be caused by individual differences among diabetic patients, since the shape and size of HSA could be modified significantly in response to pH changes or other biophysical influences [1].

We discovered less number of glycation sites in the GA from diabetic patient than the one from healthy person. Since we only tracked the MW change of 162 Da as the result of Amadori rearrangement to indicate the glycation sites during data processing, it was possible that advanced glycation end products were formed on the diabetic patient’s GA and overlooked by the LC/MS/MS approach. Caution should be taken when study the change of glycation sites from healthy person to diabetic patient and use it as an indicator of glycemic control because of the diversity and variation.

### 2.3. Incorporation Ratio of Glucose to Albumin

Glycation affects the conformation and function of HSA by occupying lysine residues. Where and how many glycation occurred may determine the degree of HSA disfuntion. It raises the importance of study incorporation ratio of glucose to albumin in addition to glycation site study. The definition of incorporation ratio here is the average number of glucose molecules attached to each albumin molecule. We presented a simple and efficient tool to estimate the incorporation ratio using MALDI-TOF-MS. With internal standard calibration, MALDI-TOF-MS is widely used to obtain the relatively accurate average MW of glycoconjugates. By calculating the MW shift from GA to NGA, the incorporation ratio of glucose to albumin could be deduced as:

IR = [MW_GA_ − MW_NGA_]/162

where 162 is the MW increase of each glucose attached to albumin.

**Figure 5:**
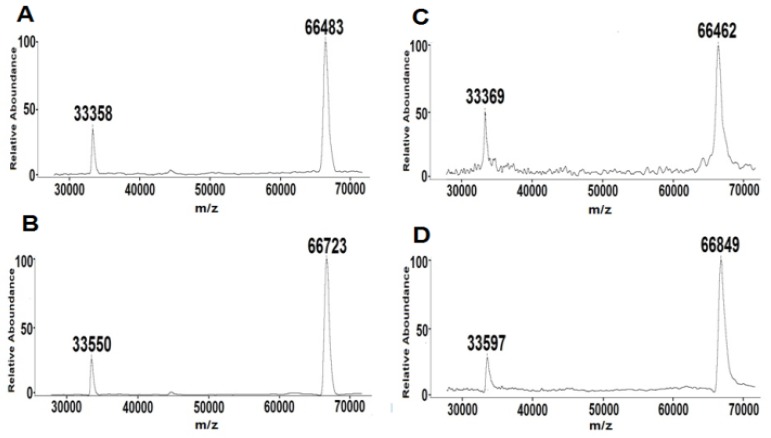
MALDI-TOF-MS analysis of serum albumin. (**A**) NGA from healthy person. (**B**) GA from healthy person. (**C**) NGA from diabetic patient. (**D**) GA from diabetic patient.

The MALDI-TOF-MS results were shown in Figure 5. The incorporation ratio of healthy person was calculated as 2.0, whereas diabetic patient was 2.5. Although the serum GA level elevated significantly from healthy person to diabetic patient, the incorporation ration only increased slightly. Our hypothesis is that glucose preferred to attack unmodified HSA instead of already glycated HSA. Further study will rely on MS analysis of detailed incorporation ratio distribution change [27] to figure out the dynamics of HSA glycation.

## 3. Experimental

### 3.1. Materials

Endoproteinase Glu-C (sequencing grade) was purchased from Roche (Mannheim, Germany) and Trypsin Gold (MS grade) was from Promega (Madison, WI, USA). TSK-gel Boronate-5PW column (10 µm, 75 × 7.5 mm I.D.) was obtained from Tosoh (Tokyo, Japan) and BetaBasic C18 reversed phase capillary LC column (5 µm, 100 × 0.32 mm I.D.) from Thermo Scientific (Barrington, IL, USA). Centricon YM-10 centrifugal filter devices [0.5 mL, 10 kDa molecular weight cut-off (MWCO)] and dialysis membranes (500-1000 Da MWCO) were purchased from Millipore (Billerica, MA, USA) and Spectrum Laboratories, (Rancho Dominguez, CA, USA), respectively. All SDS-PAGE reagents were purchased from Bio-Rad (Hercules, CA, USA). All other chemicals and reagents with best available grade were purchased from Sigma-Aldrich (St. Louis, MO, USA) and Fisher Scientific (Morris Plains, NJ, USA).

### 3.2. Sample Preparation

Serum samples were obtained from a patient with type 2 diabetes and an euglycemic healthy person, respectively. Samples were kept frozen until use. HSA was extracted from serum using PEG precipitation method described by Mashiba and coworkers [13]. Fifty mg of PEG 6000 was added to a 500 µL portion of serum. The samples were mixed completely by stirring and then centrifuged at 2000 ×g for 5 min. Globulin proteins in the precipitates were discarded and the supernatant containing HSA was transferred to a 1.5 mL Eppendorf tube.

### 3.3. Affinity Chromatography

A TSK-gel Boronate-5PW column was used to separate GA from NGA according to the procedure reported by Yasukawa [15]. The affinity chromatography was performed on LC-20AT HPLC system (Shimadzu, Tokyo, Japan). The column was equilibrated with mobile phase A (250 mM ammonium acetate containing 50 mM magnesium chloride and 5% ethanol, pH 8.5) at flowrate of 1 mL/min. Then 100 µL of supernatant from the precipitation step was loaded to the column. NGA was eluted with mobile phase A. Then mobile phase B (200 mM sorbitol, 100 mM Tris and 50 mM EDTA-2Na, pH 8.5) was pumped through the column until GA was completely eluted. The chromatography was monitored with UV detector at 280 nm. Peaks corresponding to NGA and GA were collected, dialyzed (500–1000 Da MWCO) against water 4 °C, and then lyophilized.

### 3.4. SDS-PAGE Analysis

SDS-PAGE was performed using Mini Format 1-D electrophoresis system (Bio-Rad, CA, USA) to evaluate the purity of NGA and GA. The handcasted gel was consisted of resolving gel (10% acrylamide, 2.7% *N*′*N*′-bis-methylene acrylamide) and stacking gel (4% acrylamide, 2.7% N′N′-bis-methylene acrylamide). Protein samples were denatured at 95 °C for 5 min in 2X treatment buffer (0.125 M Tris-HCl, 4% SDS, 20% glycerol, 10% 2-mercaptoethanol, pH 6.8). After cooled to room temperature, approximate 2 µg of each sample was loaded on to the surface of stacking gel. Electrophoresis was run at 200 V for 50 min. Proteins were visualized by Coomassie brilliant blue R250 staining. 

### 3.5. Enzymatic Digestion

NGA and GA samples were incubated with denaturing buffer (0.5 M Tris-HCl, 2.75 mM EDTA, 6 M guanidine-HCl, pH 8.1) at 100 °C for 15 min. After cooled to room temperature, 6 μL of 1 M dithiothreitol solution was added and incubated at 37 °C for 30 min, followed by the addition of 10 μL of 1 M iodoacetamide and further incubation in the dark at room temperature for 1 h. The reduced and alkylated samples were desalted using Centricon YM-10 centrifugal filter devices (10 kDa MWCO) and then vacufuged to dryness. Each desalted sample was reconstituted with 19 μL of 25 mM ammonium bicarbonate solution and then aliquoted into two vials. Enzymatic digestion was carried out by incubation with endoproteinase Glu-C and trypsin (1:25, w/w) at 37 °C overnight, respectively.

### 3.6. LC/MS/MS Analysis

LC/MS/MS analysis was performed on LCMS-IT-TOF mass spectrometer coupled with 2D nanoLC system (Shimadzu, Tokyo, Japan). Mobile phase A was 0.1% formic acid in 2% aqueous acetonitrile and mobile phase B was 0.1% formic acid in 98% acetonitrile. Sample injection and on-line desalting were carried out using a C18 trap column with 5% mobile phase B at flowrate of 30 μL/min delivered by a LC-20AB pump. The flow line was then switched into a BetaBasic C18 separation column (5 µm, 100 × 0.32 mm I.D.) and the peptides were eluted at flowrate of 5 μL/min by two nanoLC pumps using the following two linear gradients: 0–65 min, 5–50% mobile phase B; 65–75 min, 50–95% mobile phase B.

The ESI-MS/MS was performed in the positive ion mode and instrument parameters were set as the following: Interface voltage, +3.6 kV; nebulizing gas flowrate, 0.5 L/min; CDL temperature 200 °C; heating block temperature, 200 °C; detector voltage, 1.75 kV. Scan range was 350–1300 for MS and 100–1500 for MS/MS.

### 3.7. Data Processing 

List of theoretical molecular weights (MWs) for enzymatic digested HSA peptides was generated using PeptideMass web tool at ExPASy. Chemical formulas corresponding to these peptides were then deduced and input to the MetID software (Shimadzu). Chemical formula C_0_H_0_ was input as parent formula for unmodified peptides, while formula C_6_H_10_O_5_, which reflected one glucose incorporated to peptides, was input for glycated peptides. A maximum error of 25 ppm was permitted for peak searching when mapping experimental MS peaks to predicted peptide MWs.

### 3.8. MALDI-TOF-MS Analysis

The intact NGA and GA samples were analyzed on Axima Performance MALDI-TOF mass spectrometer (Shimadzu). A mixture of peptides with known MWs was used as internal standards to calibrate the instrument. The MW profile of each sample was obtained in the positive ion and linear mode.

## 4. Conclusions

We have demonstrated comprehensive methods for characterize HSA glycation *in vivo*. The serum GA level, glycation sites, and incorporation ratio of glucose to HSA were analyzed using chromatography and MS approaches. IT-TOF MS was proved to be a sensitive technique for glycation sites determination. The study of glycation in euglycemic people has equivalent importance as that in diabetic patients because GA was not only a potential indicator for glycemic control, but also was a carrier for drug targeting and implicated in many other severe diseases [28].

## Figures and Tables

**Table 1:** ESI-IT-TOF-MS fingerprinting of tryptic peptides of GA from healthy person.

Position	Peptide sequence	*m*/*z* calculated	*m*/*z* measured	error (ppm)
T2-3	SEVAHRFK	487.2643	487.2728	17.38
T3-4	FKDLGEENFK	409.5399	409.5366	−8.08
T4	DLGEENFK	476.2245	476.2165	−16.88
T5	ALVLIAFAQYLQQCPFEDHVK	830.7665	830.7538	−15.3
T6	LVNEVTEFAK	575.3111	575.3013	−17.11
T8	SLHTLFGDK	509.2718	509.2650	−13.4
T9	LCTVATLR	467.2629	467.2547	−17.65
T10	ETYGEMADCCAK	717.7703	717.7604	−13.86
gT10-11	ETYGEMADCCAKQEPER	745.9661	745.9593	−9.17
T12-13	NECFLQHKDDNPNLPR	666.3146	666.3077	−10.42
T14	LVRPEVDVMCTAFHDNEETFLK	884.0928	884.0804	−14.08
T15-16	KYLYEIAR	528.2978	528.2894	−15.98
T16	YLYEIAR	464.2504	464.2443	−13.05
T17-18	RHPYFYAPELLFFAK	633.6699	633.6621	−12.34
T18	HPYFYAPELLFFAK	581.6362	581.6272	−15.5
T18-19	HPYFYAPELLFFAKR	633.6699	633.6621	−12.34
gT24-26	DEGKASSAKQR	669.8284	669.8179	−15.71
gT28-29	CASLQKFGER	679.3245	679.3377	19.49
T29	FGER	508.2514	508.2404	−21.69
T30-31	AFKAWAVAR	510.2929	510.2822	−20.96
gT32-34	LSQRFPKAEFAEVSK	633.6668	633.6622	−7.25
T33-34	FPKAEFAEVSK	626.8322	626.8340	2.8
gT33-34	FPKAEFAEVSK	472.2415	472.2353	−13.19
T34	AEFAEVSK	440.7242	440.7256	3.23
T34-35	AEFAEVSKLVTDLTK	550.9698	550.9661	−6.74
gT34-35	AEFAEVSKLVTDLTK	604.9874	604.9807	−11.11
T36	VHTECCHGDLLECADDR	696.2840	696.2741	−14.26
gT37-38	ADLAKYICENQDSISSK	701.9965	701.9827	−19.71
T38	YICENQDSISSK	722.3247	722.3142	−14.48
T39-40	LKECCEKPLLEK	516.2704	516.2639	−12.66
T40	ECCEKPLLEK	653.3125	653.3032	−14.24
T41	SHCIAEVENDEMPADLPSLA ADFVESK	992.1197	992.1038	−16.0
T44	DVFLGMFLYEYAR	812.3974	812.3844	−16.04
T45-46	RHPDYSVVLLLR	489.9525	489.9460	−13.34
T46	HPDYSVVLLLR	437.9188	437.9120	−15.6
gT47-48	LAKTYETTLEK	486.9240	486.9212	−5.71
T50	VFDEFKPLVEEPQNLIK	682.3700	682.3583	−17.11
gT50	VFDEFKPLVEEPQNLIK	736.3876	736.3848	−3.78
T51	QNCELFEQLGEYK	829.3800	829.3642	−19.01
gT53-54	YTKK	701.3716	701.3727	1.57
T54-T55	KVPQVSTPTLVEVSR	547.3174	547.3109	−11.94
T55	VPQVSTPTLVEVSR	756.4250	756.4124	−16.7
gT56-57	NLGKVGSK	482.7691	482.7736	9.29
T57-59	VGSKCCKHPEAK	700.8423	700.8282	−20.11
T61	MPCAEDYLSVVLNQLCVLHEK	840.0761	840.0623	−16.47
gT62-64	TPVSDRVTKCCTESLVNR	762.0351	762.0489	18.1
T64	CCTESLVNR	569.7526	569.7459	−11.79
T65	RPCFSALEVDETYVPK	637.6487	637.6394	−14.65
T66	EFNAETFTFHADICTLSEK	754.0124	753.9980	−19.11
gT68-70	QIKKQTALVELVK	554.0012	553.9944	−12.31
T69-70	KQTALVELVK	564.8530	564.8464	−11.65
gT69-70	KQTALVELVK	645.8794	645.8683	−17.18
T70	QTALVELVK	500.8055	500.7996	−11.78
gT73-74	EQLKAVMDDFAAFVEK	668.3274	668.3174	−15.03
T74	AVMDDFAAFVEK	671.8210	671.8094	−17.3
gT74-75	AVMDDFAAFVEKCCK	651.6195	651.6146	−7.47
gT76-77	ADDKETCFAEEGK	554.5664	554.5763	17.83
T78-79	KLVAASQAALGL	571.3506	571.3419	−15.24
T79	LVAASQAALGL	507.3031	507.2958	−14.44

**Table 2:** Glycation sites on GA from healthy person and diabetic patient.

Subject	Glycation sites
healthy person	Lys-64, Lys-93*, Lys-190 or Lys-195*, Lys-205*, Lys-225*, Lys-233, Lys-262, Lys-274, Lys-281, Lys-323*, Lys-351, Lys-378, Lys-413*, Lys-432*, Lys-475, Lys-525, Lys-526*, Lys-545, Lys-557*, Lys-564*, Lys-573 or Lys-574*
diabetic patient	Lys-64, Lys-190*, Lys-199, Lys-225*, Lys-233, Lys-240*, Lys-274, Lys-281, Lys-281 or Lys-286, Lys-286*, Lys-317, Lys-323*, Lys-372*, Lys-413*, Lys-475, Lys-525, Lys-557 or Lys-560 or Lys-564*

* The glycation sites are not reported previously.

## Supplementary Materials

Supplementary materials can be accessed at: http://www.mdpi.com/1420-3049/17/8/8782/s1.
