# Supplementary material for: Investigation of Non-Enzymatic Glycosylation of Human Serum Albumin Using Ion Trap-Time of Flight Mass Spectrometry
Source: Molecules. 2012 Jul 25;17(8):8782–94. doi: 10.3390/molecules17088782 (PMC6268088; doi:10.3390/molecules17088782)
Supplement: Supplementary file 1 [file molecules-17-08782-s001.pdf]

Supplementary Materials

Table S1. Peptide fingerprinting of GA from healthy person.

		1	7	17	27
		DAHKSE	VAHRFKDLGE	ENFKALVLIA	FAQYLQQCPF
37	47	57	67	77	87
EDHVKLVNEV	TEFAKTCVAD	ESAENCDKSL	HTLFGDKLCT	VATLRETYGE	MADCCAKQEP
97	107	117	127	137	147
ERNECFLQHK	DDNPNLPRLV	RPEVDVMCTA	FHDNEETFLK	KYLYEIARRH	PYFYAPELLF
157	167	177	187	197	207
FAKRYKAAFT	ECCQAADKAA	CLLPKLDELR	DEGKASSAKQ	RLKCASLQKF	GERAFKAWAV
217	227	237	247	257	267
ARLSQRFPKA	EFAEVSKLVT	DLTKVHTECC	HGDLLECADD	RADLAKYICE	NQDSISSKLK
277	287	297	307	317	327
ECCEKPLLEK	SHCIAEVEND	EMPADLPSLA	ADFVESKDVC	KNYAEAKDVF	LGMFLYEYAR
337	347	357	367	377	387
FAKRYKAAFT	LRLAKTYETT	LEKCCAAADP	HECYAKVFDE	FKPLVEEPQN	LIKQNCELFE
397	407	417	427	437	447
ARLSQRFPKA	LLVRYTKKVP	QVSTPTLVEV	SRNLGKVGSK	CCKHPEAKRM	PCAEDYLSVV
457	467	477	487	497	507
ECCEKPLLEK	TPVSDRVTKC	CTESLVNRRP	CFSALEVDET	YVPKEFNAET	FTFHADICTL
517	527	537	547	557	567
RHPDYSVVLL	TALVELVKHK	PKATKEQLKA	VMDDFAAFVE	KCCKADDKET	CFAEEGKKLV
577 585					
AASQAALGL					

Amino acid code letters in red are from tryptic digestion and letters underlined are from Glu-C digestion. Total sequence coverage is 88.2%.

Table S2. Peptide fingerprinting of GA from diabetic patient.

		1	7	17	27
		DAHKSE	VAHRFKDLGE	ENFKALVLIA	FAQYLQQCPF
37	47	57	67	77	87
EDHVKLVNEV	TEFAKTCVAD	ESAENCDKSL	HTLFGDKLCT	VATLRETYGE	MADCCAKQEP
97	107	117	127	137	147
ERNECFLQHK	DDNPNLPRLV	RPEVDVMCTA	FHDNEETFLK	KYLYEIARRH	PYFYAPELLF
157	167	177	187	197	207
FAKRYKAAFT	ECCQAADKAA	CLLPKLDELR	DEGKASSAKQ	RLKCASLQKF	GERAFKAWAV
217	227	237	247	257	267
ARLSQRFPKA	EFAEVSKLVT	DLTKVHTECC	HGDLLECADD	RADLAKYICE	NQDSISSKLK
277	287	297	307	317	327
ECCEKPLLEK	SHCIAEVEND	EMPADLPSLA	ADFVESKDVC	KNYAEAKDVF	LGMFLYEYAR
337	347	357	367	377	387
RHPDYSVVLL	LRLAKTYETT	LEKCCAAADP	HECYAKVFDE	FKPLVEEPQN	LIKQNCELFE
397	407	417	427	437	447
QLGEYKFQNA	LLVRYTKKVP	QVSTPTLVEV	SRNLGKVGSK	CCKHPEAKRM	PCAEDYLSVV
457	467	477	487	497	507
LNQLCVLHEK	TPVSDRVTKC	CTESLVNRRP	CFSALEVDET	YVPKEFNAET	FTFHADICTL
517	527	537	547	557	567
SEKERQIKKQ	TALVELVKHK	PKATKEQLKA	VMDDFAAFVE	KCCKADDKET	CFAEEGKKLV
577 585					
AASQAALGL					

Amino acid code letters in red are from tryptic digestion and letters underlined are from Glu-C digestion. Total sequence coverage is 77.6%.
